# Endoplasmic reticulum stress disrupts lysosomal homeostasis and induces blockade of autophagic flux in human trophoblasts

**DOI:** 10.1038/s41598-019-47607-5

**Published:** 2019-08-07

**Authors:** Akitoshi Nakashima, Shi-Bin Cheng, Tae Kusabiraki, Kenichiro Motomura, Aiko Aoki, Akemi Ushijima, Yosuke Ono, Sayaka Tsuda, Tomoko Shima, Osamu Yoshino, Haruhiko Sago, Kenji Matsumoto, Surendra Sharma, Shigeru Saito

**Affiliations:** 1 Department of Obstetrics and Gynecology, University of Toyama, 2630 Sugitani, Toyama, 930-0194 Japan; 2 Departments of Pediatrics, Women and Infants Hospital of Rhode Island, Warren Alpert Medical School of Brown University, 101 Dudley street, Providence, RI 02905 USA; 3 Department of Allergy and Clinical Immunology, National Research Institute for Child Health and Development, 2-10-1 Okura, Setagaya-ku, 157-8535 Tokyo Japan; 4 Department of Obstetrics and Gynecology, Kitasato University School of Medicine, 1-15-1 Kitazato, Minami, Sagamihara, Kanagawa 252-0374 Japan; 5 Center for Maternal-Fetal, Neonatal and Reproductive Medicine, National Center for Child Health and Development, 2-10-1 Okura, Setagaya-ku, 157-8535 Tokyo Japan

**Keywords:** Protein translocation, Exocytosis, Macroautophagy

## Abstract

Pregnancy is a stress factor culminating into mild endoplasmic reticulum (ER) stress, which is necessary for placental development. However, excessive or chronic ER stress in pre-eclamptic placentas leads to placental dysfunction. The precise mechanisms through which excessive ER stress impacts trophoblasts are not well understood. Here, we showed that ER stress reduces the number of lysosomes, resulting in inhibition of autophagic flux in trophoblast cells. ER stress also disrupted the translocation of lysosomes to the surface of trophoblast cells, and inhibited lysosomal exocytosis, whereby the secretion of lysosomal-associated membrane protein 1 (LAMP1) into culture media was significantly attenuated. In addition, we found that serum LAMP1 and beta-galactosidase levels were significantly decreased in pre-eclampsia patients compared to normal pregnant women, potentially indicating lysosomal dysfunction through ER stress in pre-eclamptic placentas. Thus, we demonstrated that excessive ER stress essentially disrupts homeostasis in trophoblasts in conjunction with autophagy inhibition by lysosomal impairment.

## Introduction

Pre-eclampsia (PE), which is diagnosed upon the emergence of hypertension and proteinuria in pregnant women after 20 weeks of gestation, is a human pregnancy-specific disorder and a leading cause of maternal and neonatal morbidity and mortality^1,2^. PE, affecting nearly 5–8% of all pregnant women worldwide, is a risk factor for chronic diseases later in life such as cardiovascular disease, diabetes mellitus, kidney disease, and chronic hypertension^3–6^. While the etiology of PE still remains elusive, systematic symptoms of PE are thought to be associated with poor placentation-induced “placental dysfunction”. A number of pathological paradigms contribute to poor placentation at the maternal-fetal interface, such as ischemia/hypoxia, inflammation, oxidative stress, and endoplasmic reticulum (ER) stress. Poor placentation also induces an increase in anti-angiogenic factors, such as soluble fms-like tyrosine kinase 1 (sFLT1) and soluble endoglin (sENG)^7–9^, leading to imbalanced angiogenesis and endothelial dysfunction.

We have reported that failure to induce autophagy in trophoblasts contributes to gestational hypertension and poor placentation, *in vitro* and *in vivo*, which are a part of the etiology of PE^10–12^. Autophagy is a bulk degradation system present in cells that maintains cellular homeostasis by producing energy, or eliminating misfolded or aggregated proteins which are not otherwise degraded by the ubiquitin-proteasome system^13^. Lysosomes are synthesized in the ER and bud from the Golgi apparatus, and are fundamental for autophagy. Lysosomes function in the final step of autophagy when they fuse with autophagosomes to form autolysosomes, and their inner contents are degraded by lysosomal hydrolases^14^. Thus, functional lysosomes regulate an array of cellular functions, including not only autophagy, but also cell cycle, cell growth and macromolecular biogenesis^15^.

ER plays a central role in protein synthesis, structural modification, and correct folding of secreted and membrane proteins, in all eukaryotic cells^16^. Once misfolded or unfolded proteins accumulates in the lumen of ER, unfolded protein response (UPR) is activated which increases protein-folding and protein-degradation activity. UPR allows cells to overcome ER stress, but overwhelming protein aggregation and excessive ER stress cannot always be countered by intrinsic cellular mechanisms, resulting in cell degeneration and programmed cell death^17^. Excessive ER stress in the placenta has been documented as a pivotal factor contributing to the pathophysiology of PE or fetal growth restriction (FGR)^18–21^, suggesting that pathological placentas are under “terminal overwhelming” situation. Recent observations have revealed an association between ER stress and autophagy in that ER stress can either stimulate or inhibit autophagy^22^. However, consequential ER stress and its effect on autophagy in trophoblasts are still poorly addressed^23^.

Here we show that excessive ER stress induced by brefeldin A (BFA) or TM, impaired lysosomal function and consequently inhibited autophagic flux through increasing autophagosomes and decreasing autolysosomes. Additionally, the expression levels of lysosomal-associated membrane protein 1 (LAMP1) and beta-galactosidase (b-gal), a lysosomal hydrolytic enzyme, in human serum were significantly reduced in PE patients compared to normal pregnant women, suggesting that serum LAMP1 or b-gal can be used as a marker for ER stress-induced lysosomal impairment in the human placenta.

## Results

### ER stress inhibited autophagic flux by blocking autophagosome-lysosome fusion in HchEpC1b trophoblast cells

**Figure 1:**
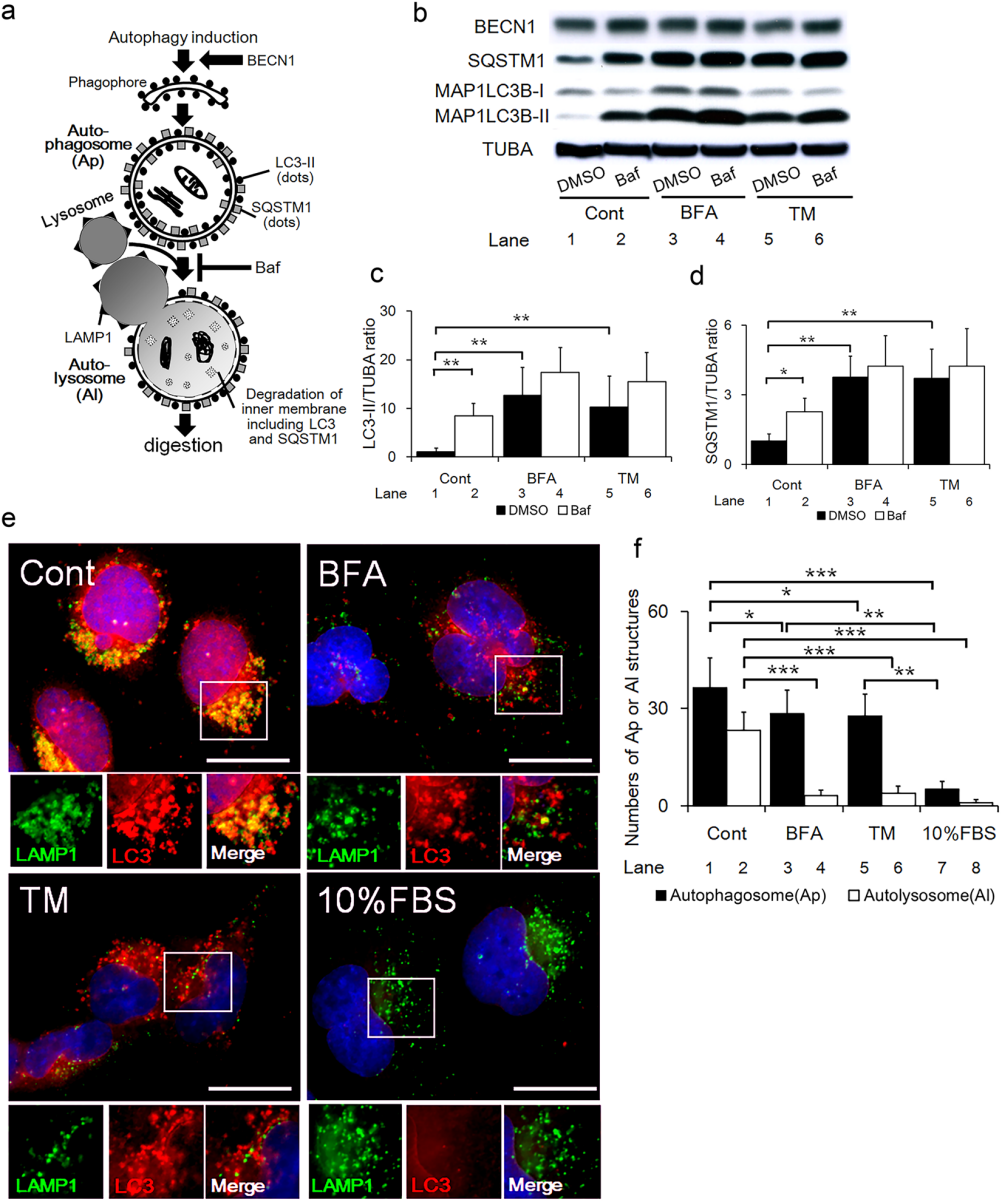
ER stress inhibited autophagic flux by blocking autophagosome-lysosomal fusion. (**a**) Schematic showing the steps of maturation from phagophore to autolysosome in the autophagy machinery. MAP1LC3B-II (also known as LC3-II) and SQSTM1 (also known as p62) are bound to autophagosome membranes. Autolysosomes are generated by the fusion of autophagosomes with lysosomes expressing LAMP1. The inner membrane including MAP1LC3B-II and SQSTM1 is digested in the autolysosomes. (**b**) Western blots of HchEpC1b cells cultured with 500 ng/ml of brefeldin A (BFA) or 500 ng/ml of tunicamycin (TM) for 24 h, with or without 10 nM of bafilomycin A1 (Baf) treatment for 2 h at the end of culture, are shown as follows: BECN1, SQSTM1, MAP1LC3B (LC3), and TUBA. The graph shows the expression levels of MAP1LC3B-II (**c**) or SQSTM1 (**d**) in HchEpC1b cells, cultured with BFA or TM in the presence (white bars) or absence (black bars) of Baf. Expression was normalized to TUBA levels. (**e**) Representative panels showing the merged images of anti-LAMP1 staining (green), anti-MAP1LC3B staining (LC3, red) and nuclear staining (DAPI, blue) in HchEpC1b cells. The smaller images show the anti-LAMP1, anti-LC3, and the merged images of the area surrounded by white lines in the large images. For this experiment, the cells were cultured under serum free condition with 50 μM of chloroquine for 2 h at the end of culture, with DMSO (Cont), 500 ng/ml BFA or 500 ng/ml TM. As a negative control, HchEpC1b cells were cultured in RPMI1640 medium with 10% FBS without chloroquine. (**f**) The graph shows the average numbers of autophagosomes (Ap, black bars) or autolysosomes (Al, white bars) in HchEpC1b cells, which were cultured with DMSO (Cont), 500 ng/ml BFA, 500 ng/ml TM or 10% FBS. Data is expressed as the mean ± S.D. *p < 0.05, **p < 0.01, ***p < 0.001. Scale bars: 20 μm.

We first examined if ER stress or oxidative stress, which often accompanies the pathophysiology of PE, affects autophagy in the 1st-trimester extravillous trophoblast (EVT) cell line, HchEpC1b cells. To evaluate the autophagy pathway, we examined the expression levels of beclin1 (BECN1, an initiator of autophagosomes), microtubule-associated protein 1 light chain 3 beta-II (MAP1LC3B-II, an autophagosome marker, also known as LC3-II), and sequestosome1 (SQSTM1, a substrate of autophagy, also known as p62) (Fig. 1a). ER stress inducers, BFA and TM, increased the expression of MAP1LC3B and SQSTM1, but not BECN1, compared to control cells treated with DMSO (Fig. 1b, lane 1, 3 and 5). On the other hand, hydrogen peroxide, an oxidative stress inducer, did not increase MAP1LC3B-II expression (Supplemental Fig. 1, lane 1 and 2, 3 and 4).

To next estimate the autophagic flux, the ratio of MAP1LC3B-II to TUBA was compared in the cells treated with BFA or TM, in the presence of bafilomycin A1 (Baf), an inhibitor of autophagosome-lysosome fusion (Fig. 1b, lane 2, 4 and 6). MAP1LC3B-II/TUBA ratio was significantly increased in control cells treated with Baf (Fig. 1c, lane 1 and 2), showing the activation of autophagy flux. In contrast, the MAP1LC3B-II/TUBA ratio was not significantly different between Baf treated and untreated cells, when they were co-treated with BFA or TM (Fig. 1c, lane 3 and 4, 5 and 6). Similar results were obtained with the ratio of MAP1LC3B-II/MAP1LC3-B-I, another autophagy flux marker (Supplemental Fig. 2). In addition, SQSTM1, a chaperone of ubiquinated proteins of the autophagosome, which binds to autophagosomes and is degraded by autophagy pathway^24^ (Fig. 1a), was significantly accumulated in cells treated with BFA or TM (Fig. 1d, lane 1, 3 and 5). These findings indicated that ER stress increased the number of autophagosomes, as evidenced by an increase in MAP1LC3B-II levels. However, Baf treatment failed to influence ER stress-induced accumulation of SQSTM1 (Fig. 1d, lane 3, 4 and 5, 6), suggesting that ER stress is sufficiently able to block autophagic flux at the step of autophagosome-lysosomal fusion in the EVT cell line.

As shown in Fig. 1a, autophagosomes were recognized with punctate appearance of MAP1LC3B without LAMP1 co-localization. Autolysosomes were marked by the co-localization of MAP1LC3B and LAMP1. We next tested whether ER stress inhibited autophagy through impairing autophagosome-lysosome fusion. To estimate autophagosome-lysosome fusion, double immunostaining for MAP1LC3B and LAMP1 was performed in cells incubated in serum free medium. Autophagy induction under serum-free culture conditions was confirmed in HchEpC1b cells by western blotting (Supplemental Fig. 3, lane 3). On the other hand as suspected, nutrient rich conditions (10% FBS), which did not activate autophagy as demonstrated in Supplemental Fig. 3, lane 1, were used as a negative control for autophagy activation. Cells were treated with chloroquine (CHQ) to block lysosomal functions by its neutralization. Upon treatment with CHQ, although majority of MAP1LC3B dots co-localized with LAMP1 staining in control cells, only few MAP1LC3B dots co-localized with LAMP1 in cells treated with BFA or TM (Fig. 1e). The number of autophagosomes was higher in cells with ER stress than that in cells with 10% FBS (Fig. 1f, lanes 3, 5 and 7); the number of autolysosomes in cells treated with BFA or TM were equivalent to that in cells with 10% FBS (Fig. 1f, lane 4, 6 and 8). Meanwhile, the number of autolysosomes in cells with ER stress was significantly lower than that seen in controls (Fig. 1f, lane 2, 4 and 6). In addition, the number of LAMP1 dots was decreased in the cells with BFA and TM, compared to the control or 10% FBS (Supplemental Fig. 4). Thus, ER stress increased the number of autophagosomes, but not autolysosomes, suggesting the blockage of autophagosome-lysosome fusion by ER stress in HchEpC1b cells.

### ER stress suppressed the expression of LAMPs in HchEpC1b trophoblast cells

**Figure 2:**
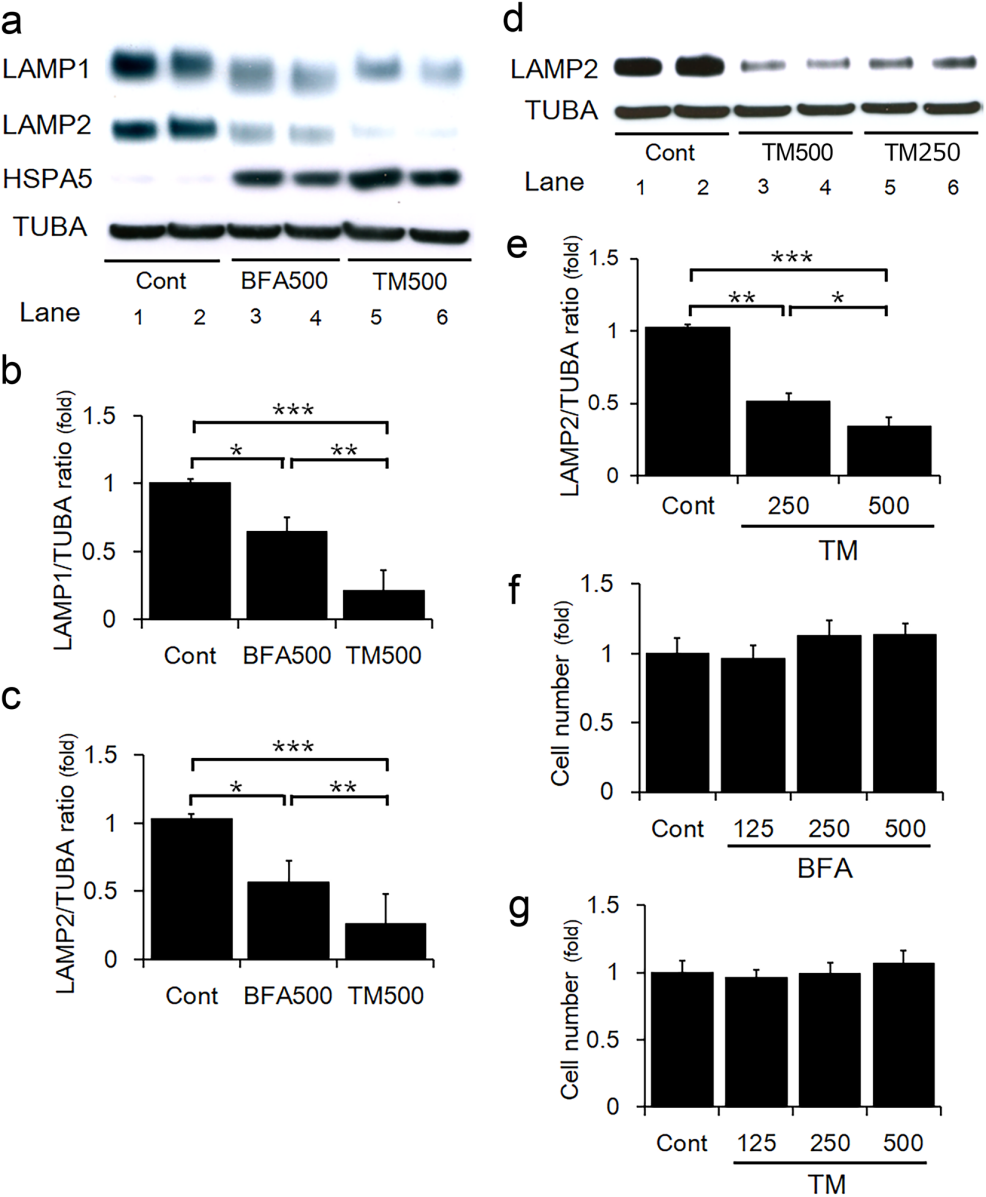
ER stress suppressed the expression of LAMPs in HchEpC1b cells. (**a**) Western blots of HchEpC1b cells, cultured with 500 ng/ml of brefeldin A (BFA) or 500 ng/ml of tunicamycin (TM) for 24 h, is shown as follows: LAMP1, LAMP2, HSPA5, and TUBA. The graph shows the expression levels of LAMP1 (**b**) or LAMP2 (**c**) in the HchEpC1b cells, cultured with BFA or TM. Expression is normalized to TUBA levels. (**d**) LAMP2 expression in HchEpC1b cells cultured with 250 or 500 ng/ml of TM for 24 h, is shown by western blotting. (**e**) The graph shows the expression levels of LAMP2 normalized with TUBA levels, in HchEpC1b cells cultured with 250 or 500 ng/ml of TM for 24 h. Cell proliferation assay was performed on HchEpC1b cells cultured with 125, 250 or 500 ng/ml of BFA (**f**) or 125, 250 or 500 ng/ml of TM (**g**) for 24 h. Data expressed as mean ± S.D. *p < 0.01, **p < 0.01, ***p < 0.001.

To assess if ER stress decreased the number of lysosomes in trophoblasts, the levels of the lysosomal proteins LAMP1 and LAMP2 were examined. LAMP1 and LAMP2 levels decreased in HchEpC1b cells in response to ER stress inducers, BFA or TM, whereas the expression of heat shock protein family A member 5 (HSPA5, also known as Bip), a marker of ER stress, was markedly increased (Fig. 2a, lane 3–6). The decrease of LAMP1 expressions by TM was more pronounced than that induced by BFA at the same concentration in HchEpC1b cells (Fig. 2b). Similar results were obtained for LAMP2 expression (Fig. 2c). A decrease in lysosomal proteins during ER stress was also observed in TCL1 cells, a 3rd-trimester trophoblast cell line (Supplemental Fig. 5a–c). In addition, thapsigargin (TPG) treatment, an inhibitor of endoplasmic reticulum Ca^2+^ - ATPase, of BeWo cells resulted in decrease in the LAMP1 protein content, particularly at 100 nM dose (Supplemental Fig. 5d). In parallel, TPG increased relative protein band intensities of HSPA5, DDIT3, and SQSTM1. The increase of SQSTM1, which indicated autophagy inhibition, was accompanied in cells with 100 nM, but not 5 nM of TPG. However, oxidative stress did not affect LAMP1 expression in HchEpC1b cells (Supplemental Fig. 6). In addition, LAMP2 expression decreased in a TM dose-dependent manner (Fig. 2d, e). BFA or TM treatments did not show any inhibitory effects on cell proliferation in HchEpC1b cells at 24 h (Fig. 2f, g), suggesting that excessive ER stress downregulated lysosomal proteins, but did not influence cell growth in the trophoblast cell line.

### ER stress suppressed lysosomal functions in HchEpc1b cells

**Figure 3:**
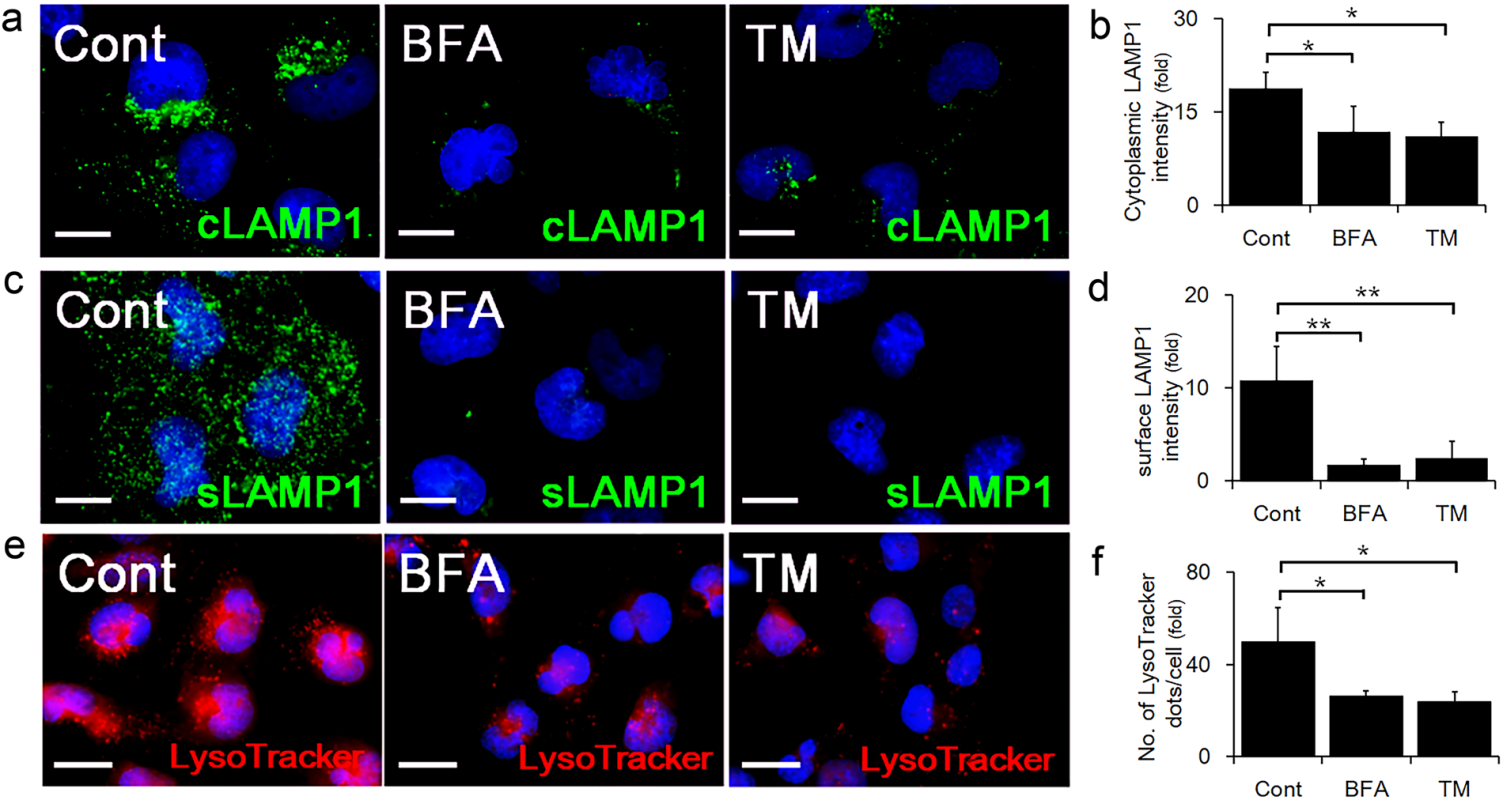
ER stress downregulated cytoplasmic and surface LAMP1. Immunocytochemical analysis showing staining of cytoplasmic LAMP1 (cLAMP1) in permeabilized HchEpC1b cells, or surface LAMP1 (sLAMP1) docked to the plasma membrane, in non-permeabilized HchEpC1b cells. (**a**) Representative panels showing the merged images of cLAMP1 (green) and nuclear staining (DAPI, blue) in HchEpC1b cells cultured with 500 ng/ml of brefeldin A (BFA) or 500 ng/ml of tunicamycin (TM) for 24 h. (**b**) The graph shows the intensity of cLAMP1 in HchEpC1b cells cultured with 500 ng/ml of BFA or 500 ng/ml of TM for 24 h. (**c**) Representative panels showing the merged images of sLAMP1 (green) and nuclear staining (DAPI, blue) in HchEpC1b cells cultured with 500 ng/ml of BFA or 500 ng/ml of TM for 24 h. (**d**) The graph shows the intensity of sLAMP1 in HchEpC1b cells cultured with 500 ng/ml of BFA or 500 ng/ml of TM for 24 h. (**e**) Acidic organelles, mainly lysosomes, are detected by LysoTracker staining. (**f**) The graph shows the number of LysoTracker-positive dots per cell in HchEpC1b cells cultured with 500 ng/ml of BFA or 500 ng/ml of TM for 24 h. Data expressed as mean ± S.D. *p < 0.05, **p < 0.01. Scale bars: 20 μm.

The lysosome in the cytoplasm is responsible for degrading misfolded and aggregated proteins, whereas the lysosome on the cell membrane surface can facilitate membrane repair or exocytosis^15^. To estimate the distribution of the lysosomes in trophoblasts, immunocytochemical and flow cytometric analyses were performed. ER stress induced by BFA or TM significantly decreased the intensity of cytoplasmic LAMP1, in the peri-nuclear areas (Fig. 3a, b). This was consistent with the result obtained by western blotting (Fig. 2a). The presence of surface LAMP1 was not detected in cells treated with BFA or TM (Fig. 3c, d). Quantitative analysis using flow cytometry on cells detached from dishes by trypsin, showed a decrease in cytoplasmic and surface LAMP1 staining in TM-treated cells (Supplemental Fig. 7a–c). Similar results were obtained for LAMP2 staining (Supplemental Fig. 7d–f). To estimate the degradation capacity of the lysosomes, the acidic organelles were detected using the LysoTracker probe that selectively accumulates in low pH cellular compartments. The number of LysoTracker-positive dots per cell was significantly lower in cells treated with BFA or TM, compared to the control (Fig. 3e, f). These findings implied that ER stress suppresses lysosomal functions by decreasing the number of lysosomes in the trophoblast cell line.

### Lack of autolysosome formation under ER stress due to diminished lysosomes in primary human trophoblasts

**Figure 4:**
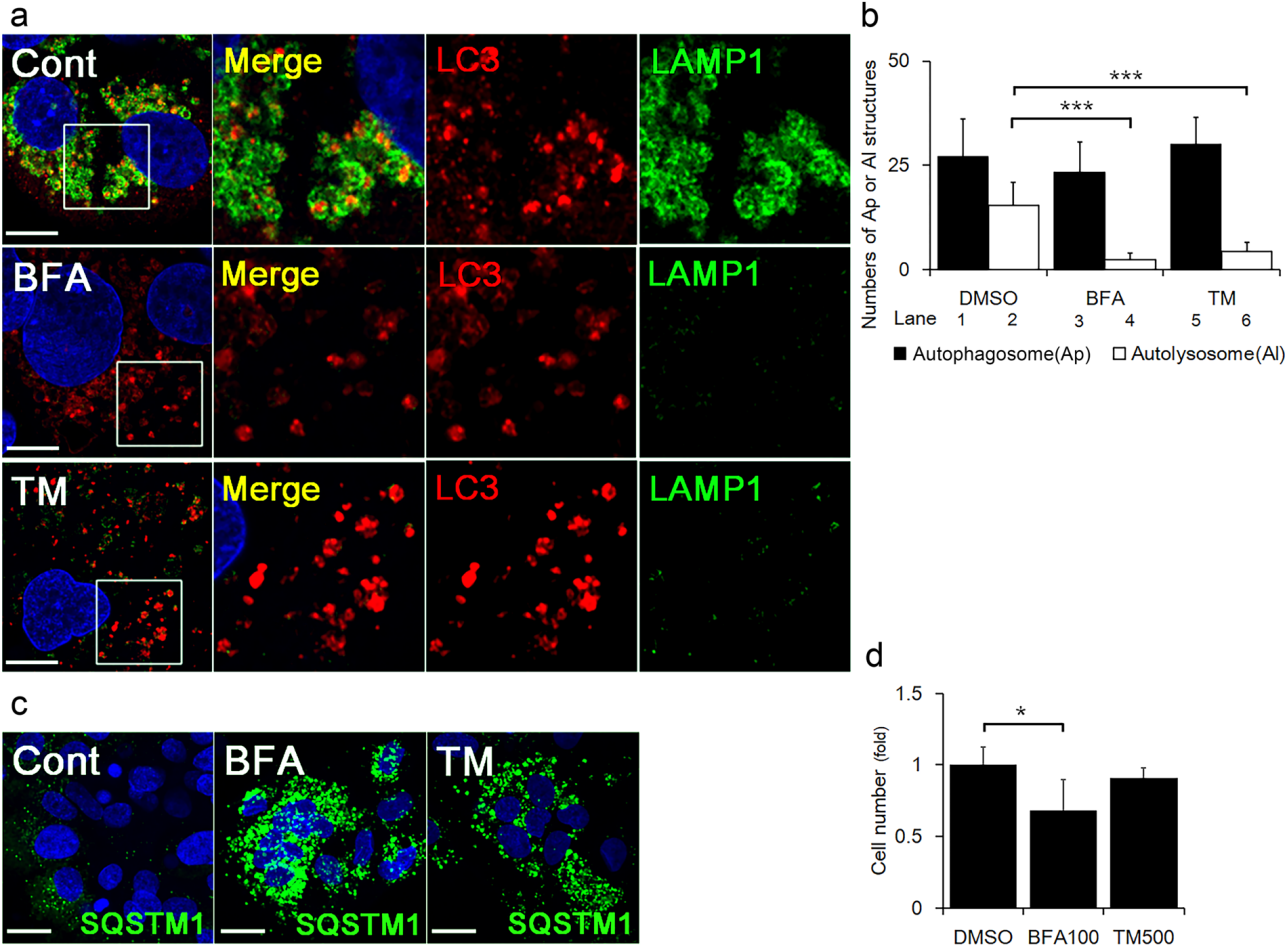
ER stress impaired lysosome function in primary human trophoblasts. (**a**) Representative panels showing the merged images of anti-LAMP1 staining (green), anti-MAP1LC3B staining (LC3, red) and nuclear staining (DAPI, blue) in primary human trophoblasts cultured with DMSO (Cont), 100 ng/ml of BFA or 500 ng/ml of TM for 24 h. The enlarged images of the area surrounded by white lines in the left images are aligned at the right as follows: merge, anti-LC3 and anti-LAMP1. (**b**) The graph shows the average numbers of autophagosomes (Ap, black bars) or autolysosomes (Al, white bars) in primary human trophoblasts cultured with DMSO (Cont), 100 ng/ml of BFA or 500 ng/ml of TM for 24 h. (**c**) Representative panels showing the merged images of anti-SQSTM1 staining (green) and nuclear staining (DAPI, blue) in primary human trophoblasts cultured with DMSO, 100 ng/ml of BFA or 500 ng/ml of TM for 24 h. (**d**) Cell proliferation assay was performed on primary human trophoblasts cultured with 100 ng/ml of BFA, or 500 ng/ml of TM for 24 h. Data expressed as mean ± S.D. *p < 0.05, ***p < 0.001. Scale bars: 10 μm.

We next examined whether ER stress induced by BFA or TM, decreased lysosomes in primary human trophoblasts. MAP1LC3B dots were spontaneously detected in some primary human trophoblasts, that were in the process of differentiation to syncytiotrophoblasts, suggesting the activation of autophagy during differentiation. As observed previously in Fig. 1e, LAMP1 expression was downregulated in primary human trophoblasts treated with BFA or TM (Fig. 4a, green). MAP1LC3B dots were surrounded by, or co-localized with LAMP1 at the peri-nuclear area in control cells (Fig. 4a, upper panels). In contrast, BFA- or TM-treated cells exhibited strong signals for MAP1LC3B dots, but only weak or undetectable signals for LAMP1 and no co-localization of MAP1LC3B dots with LAMP1 (Fig. 4a, middle and lower panels). As previously shown in Fig. 1f, ER stress also decreased the number of autolysosomes, but not autophagosomes, in primary human trophoblasts (Fig. 4b). In addition, SQSTM1 dots, which bind to aggregated proteins^24^, were significantly increased in BFA- and TM- treated cells compared to control (Fig. 4c). In this context, the accumulated SQSTM1 would bind to aggregated proteins, which may be the consequence of ER stress-induced blockage of autophagic flux in primary human trophoblasts. On the other hand, the cell numbers were lower in the cells with BFA, but not with TM than that with control (Fig. 4d). Compared with the result using the cell line (Fig. 2f,g), primary human trophoblasts differentiate and exhibit elevated susceptibility to ER stressors such as BFA. Taken together, lysosomal impairment by ER stress was observed in primary human trophoblasts.

### Lysosomal exocytosis was reduced in PE patients

**Figure 5:**
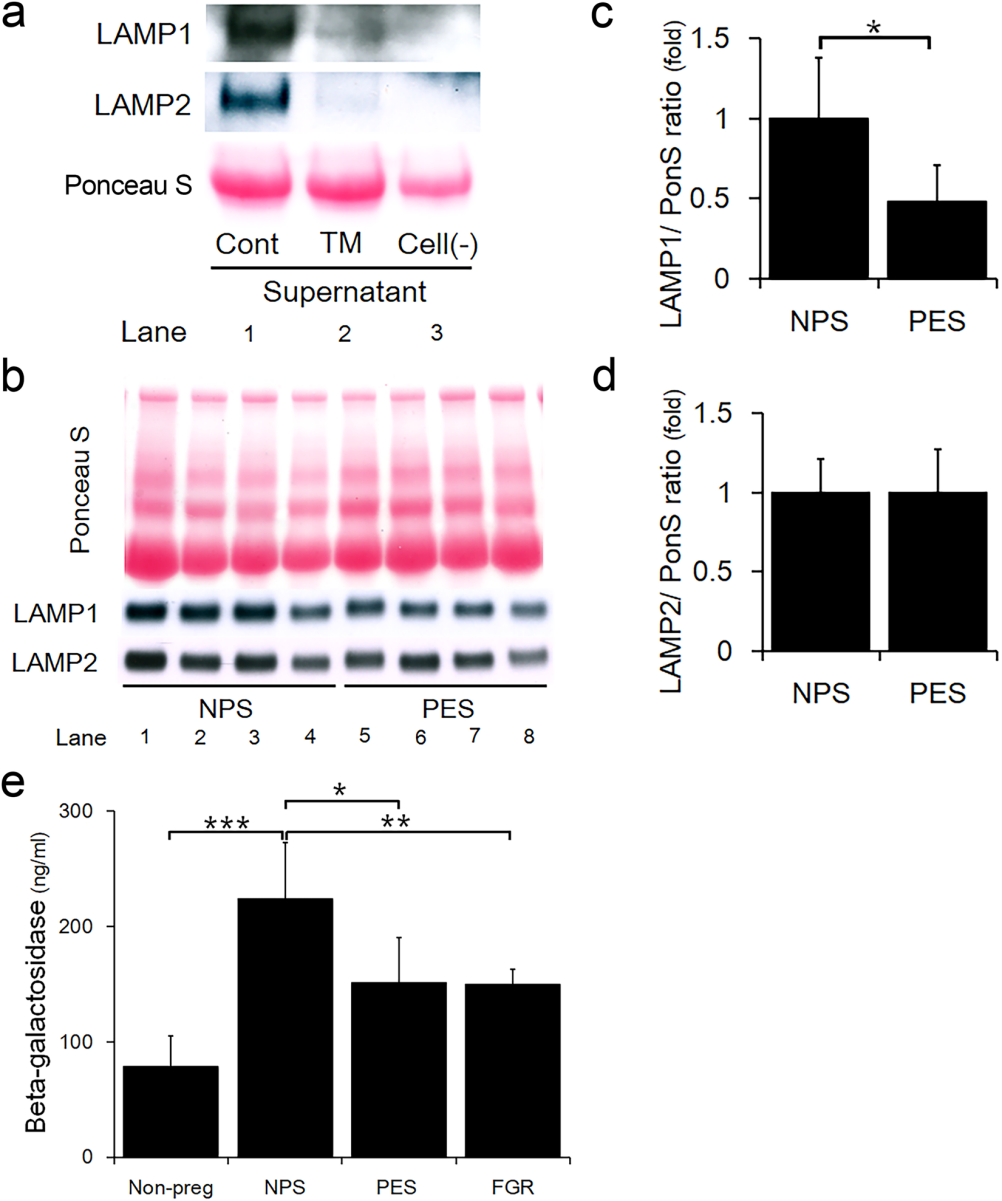
Decrease in LAMP1 expression in serum of pre-eclampsia patients. (**a**) Western blot analysis showing LAMP1 and LAMP2 expression secreted in the medium of HchEpC1b cells cultured with DMSO (Cont), or 500 ng/ml of TM for 48 h, or culture medium without cells [Cell (−)]. (**b**) Western blot analysis showing LAMP1 and LAMP2 expression levels in human serum samples. The total protein levels on the membranes estimated by Ponceau S staining. The graph shows the expression levels of LAMP1 (**c**) or LAMP2 (**d**) in the sera. Expression is normalized to Ponceau S staining levels. (**e**) The graph shows the concentration of beta-galactosidase (b-gal) in human serum samples. Data expressed as the mean ± S.D. *p < 0.05, **p < 0.01, ***p < 0.001. NPS: normal pregnant sera, PES: pre-eclampsia sera. FGR: fetal growth restriction, Non-preg: Non-pregnancy.

Recently, lysosomal exocytosis has been identified as a general mechanism for secretion in non-secretory as well as secretory cells^25,26^. As a significant decrease in surface LAMP1 was observed in the trophoblast cell line under ER stress (Fig. 3c), LAMP proteins in the culture medium were measured to evaluate lysosome-mediated exocytosis. As shown in Fig. 5a, LAMP1 and LAMP2 proteins were remarkably lower in the culture medium from TM-treated cells, compared to control cells. To examine the correlation between ER stress-mediated lysosomal impairment and pre-eclamptic patients who suffered from ER stress, we evaluated the LAMP1 and LAMP2 expression levels in human serum samples. The information of patients who provided the serum samples are shown in Table 1. LAMP1 and LAMP2 were detected in human pregnant sera (Fig. 5b). The ratio of LAMP1 to Ponceau S was significantly lower in sera from pre-eclampsia patients versus normal pregnancies (Fig. 5c). However, the ratio of LAMP2 to Ponceau S showed no difference between these two groups (Fig. 5d). Furthermore, we measured b-gal levels^27^, a lysosomal hydrolytic enzyme, in sera from women with non-pregnancy, normal pregnancy, pre-eclampsia, and FGR. As shown in Fig. 5e, the b-gal concentration was significantly higher in normal pregnant women than non-pregnant women, suggesting that the increase in the b-gal content might have been contributed by gestational tissues, including the placenta. On the other hand, the b-gal concentration of PE or FGR was significantly lower than that of NP, and there was no significant difference between PE and FGR. These results provided additional evidence for placental lysosomal dysfunctions in PE or FGR.

**Table 1:** Characteristics of human subjects.

	Non-pregnancy	Normal pregnancy (NP)	Preeclampsia (PE)	FGR	*p-*value
Subjects numbers	7	8	7	7	*n.s*.
Maternal Age (year)	31.3 ± 4.3	32.9 ± 3.0	34.3 ± 3.3	34.9 ± 2.5	*n.s*.
Primiparity (n)	6	4	5	4	*n.s*.
Body mass index (kg/m^2^)	21.1 ± 3.1	24.4 ± 1.5	26.0 ± 2.9	23.1 ± 3.1	*n.s*.
Gestational age (weeks)	*n. a*.	34.3 ± 2.6	31.3 ± 2.3	34.9 ± 3.3	*n.s*.
Systolic blood pressure (mmHg)	*n. a*.	121.8 ± 6.1	163.0 ± 5.3	120.1 ± 6.2	*p* = 0.002
Diastolic blood pressure (mmHg)	*n. a*.	68.8 ± 5.2	96.0 ± 6.5	63.6 ± 4.2	*p* = 0.002
Birthweight (g)	*n. a*.	2641.4 ± 495.6	1331.6 ± 305.6	1421.4 ± 455.1	*p* = 0.002
New born gender (M:F)	*n. a*.	4:4	4:3	3:4	*n.s*.

## Discussion

Physiological ER stress can regulate the production of vascular endothelial growth factor A (VEGF A) in trophoblasts, which is crucial for normal placental development^28^. Autophagy also plays a central role in regulating placental homeostasis and maternal circulation^10^. Interestingly, our current findings demonstrate that excessive ER stress results in reduction of lysosomal biogenesis, accumulation of SQSTM1 and inhibition of lysosomal fusion with autophagosome, thereby blocking autophagy flux in human trophoblasts. Additionally, we clarified that ER stress-promoted lysosomal inhibition decreased the secretion of LAMP proteins from a trophoblast cell line. Notably, we develop a novel assay for quantifying secreted LAMP1 in culture media of human trophoblast cells. Using this assay, we then showed the decrease of LAMP1 and lysosomal hydrolases, b-gal, which have been previously used for measuring lysosomal exocytosis^27^, in human PE sera. This approach allowed us to determine the disruption of autophagy-lysosome axis by measuring lysosomal proteins in sera of PE patients.

**Figure 6:**
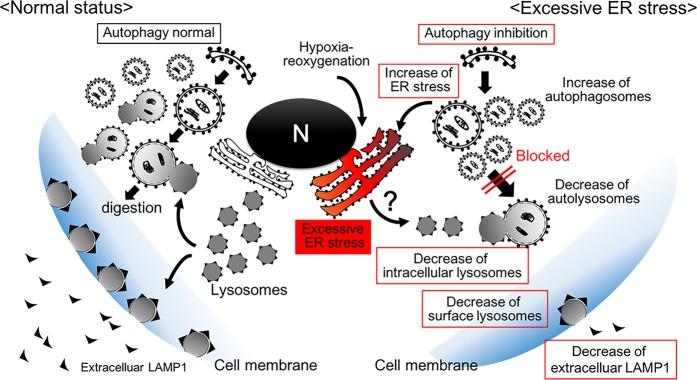
Diagram: excessive ER stress-mediated disruption of homeostasis in trophoblasts. Prospective status under normal condition, including physiological ER stress, is shown in left: lysosomes serve in the autophagy pathway for degradation, or are located on the cell surface for membrane repair or secretion. Pathological condition, including excessive ER stress, is shown in right. Hypoxia-reoxygenation stress or autophagy inhibition (Supplemental Fig. 6) potentially initiates excessive ER stress. ER stress decreases the number of intracellular lysosomes contributing to autophagy inhibition; or the number of surface lysosomes, which might be related to secretion. Excessive ER stress and autophagy inhibition could cooperatively disrupt homeostasis in trophoblasts.

ER stress has been related with the reduced production of PGF and other pathological implications of human pregnancy complications^18^. Here, we show that autophagy inhibition induces ER stress in trophoblast cells (Supplemental Fig. 8b). Thus, cross-talking between ER stress and impaired autophagy may cause poor placentation with reduced PGF production, which is also seen in placenta-specific autophagy knockout mice^10^. In Fig. 6, we proposed a model that summarizes our findings and overall hypothesis for the regulation of homeostasis in trophoblasts. Excessive or chronic ER stress impairs lysosomes, resulting in disruption of autophagy and homeostasis in trophoblasts. Assessment of the levels of LAMP1 and b-gal in sera may be used to determine if ET stress occurs in the placenta of pregnant women.

It is not clear whether autophagy inhibition affects ER stress in human trophoblasts. To address this question, HchEpC1b cells were treated with three autophagy inhibitors, wortmannin, (blocking phagophore formation) and CHQ or Baf (blocking lysosome-autophagosome fusion) (Supplemental Fig. 8a). Pharmacological autophagy inhibition increased the HSPA5 expression, but not decreased the LAMP1 expression in the trophoblast cell line (Supplemental Fig. 8b). Thus, autophagy inhibition potentially enhanced ER stress by decreasing the digestibility of unfolded or long-lived proteins in trophoblasts, and subsequently ER stress enhanced autophagy inhibition via lysosomal impairment. Thus, ER stress and impaired autophagy are intertwined with each other, leading to vicious cycle in the pathophysiology of PE (Fig. 6).

In this study, ER stress, but not oxidative stress, mediated autophagy inhibition in human trophoblasts. Hypoxia-reoxygenation increases expression of HSPA5, an indicator of ER stress, as well as HSPB1 and HSP90, indicators of oxidative stress, in cultured villous explants^20,29^. As TPG mimics physiological ER stress in trophoblasts^18^, TPG-induced ER stress results in reduction of LAMP1 and accumulation of SQSTM1 in BeWo cells (Supplemental Fig. 5d). Physiological low oxygen tension-mediated autophagy activation enhances trophoblast invasion that is required for normal placentation. On the other hand, excessive hypoxic stress inhibits trophoblast invasion in autophagy-deficient trophoblast cell lines^11,12,30^. As hypoxia-reoxygenation is known to be more harmful than hypoxia alone in cardiac cells^31^, it potentially impedes trophoblast invasion via ER stress-mediated autophagy inhibition. Prior studies reveal that lipid-induced ER stress inhibits autophagosome-lysosomal fusion in hepatocytes^32^, and similarly, saturated fatty acids disrupt autophagy flux, leading to inhibition of trophoblast invasion^33^. While the mechanisms still remain unclear, ER stress and autophagy inhibition cooperatively disrupt homeostasis in trophoblasts via impairment of lysosomes, which might play a central role in placenta-related complications such as PE and FGR.

In addition, a recent study shows that placenta-specific autophagy knockout mice exhibit reduction of PGF mRNA in the placenta as well as PE-like features such as hypertension, suggesting that autophagy deficiency may decrease PGF production in the placenta^10^. Interestingly, placental ER stress in mice and women with PE or FGR are also associated with reduction of PGF expression^18,21,34^. Again, these data suggest that ER stress and dysregulated autophagy may synergistically contribute to pathological alternation in adverse pregnancy outcome. Recently, the role of PGF was proposed to ameliorate maternal hypertension, but not affect placental weights, in a PGF and catechol-O-methyltransferase (COMT) double knockout mouse model^35^. Taken together, PGF may serve as a therapeutic option for alleviating placental ER stress and/or impaired autophagy-promoted pathological features.

Lysosomal exocytosis, by which lysosomes are docked to the plasma membrane in a LAMP1 dependent manner, has been reported to play an important role in the release of exosomes in tumor cells^36^. Prior studies have shown that release of exosomes in the maternal circulation is gestational age-dependent in normal preganancy^37^. Exosomes derived from trophoblasts have been shown to activate autophagy in non-placental cells via transfer of miRNA^38^. Thus, exosomes released from the placenta may play a crucial role for maintaining normal pregnancy. Intriguingly, our current results have demonstrated for the first time that ER stress inhibits secretion of LAMP1 from cultured trophoblast cell line, and supportively, serum LAMP1 is significantly lower in PE patients (Fig. 5a, b, c). These findings suggest that ER stress may inhibit lysosomal exocytosis, and as a result, lead to attenuation of exosome release in trophoblasts. In line with this, reduction of placenta-derived exosomes containing placental protein 13 or syncytin-2 has been observed in PE women^39,40^. While it remains to be elucidated whether LAMP1 and b-gal are secreted as cargoes of placenta-derived exosomes, our data suggest that serum LAMP1 and b-gal may be used as biomarkers for PE pathology.

## Methods

### Ethics statement and human subjects

This study was conducted in accordance with the Declaration of Helsinki, and was approved by the Ethics Committee of University of Toyama (Approval No. 28–144), or the Ethics Committee of the National Center for Child Health and Development (Approval No. 476). All subjects provided written informed consent for this study prior to serum and tissue sampling. Serum and placental tissue samples were obtained from pregnant women from 22 to 36 weeks of pregnancy. The clinical information on pregnant women for this study is shown in Table 1. Exclusion criteria were pregnant women who had a multifetal pregnancy, were younger than 18 or older than 45 years old, had pre-pregnancy hypertension, were known as HIV, hepatitis B virus, or hepatitis C virus positive, and refused to give informed consent. Placental tissues were collected within 2 h from the time of delivery.

## Reagents

Antibodies used for western blotting or immunocytochemistry were as follows: Mouse monoclonal antibodies (Ab) - anti-BECN 1 (sc48341, Santa Cruz Biotechnology, Dallas, TX, USA), anti-DDIT3 (2895, Cell Signaling technology, Danvers, MA, USA), anti-LAMP1 (ab25630, Abcam, Cambridge, UK), anti-LAMP2 (ab25631, Abcam), anti-SQSTM1/p62 (M162-3, MBL, Nagoya, Japan), anti-α-TUBA (T8203, Sigma-Aldrich, St. Louis, MO, USA) and normal mouse IgG (sc-2025, Santa Cruz Biotechnology); Rabbit polyclonal Ab - anti-MAP1LC3B (PM036, MBL), anti-HSPA5 (3183, Cell Signaling technology) and normal rabbit IgG (sc-2027, Santa Cruz Biotechnology). The following secondary Ab were used: anti-mouse IgG-HRP conjugate (7076, Cell Signaling technology), anti-rabbit IgG-HRP conjugate (7074, Cell Signaling technology), Alexa Fluor 488 Donkey anti-mouse IgG secondary antibody (A-21202, Thermo Fisher Scientific, Waltham, MA, USA), and Alexa Fluor 555 Donkey anti-rabbit IgG secondary antibody (A-31572, Thermo Fisher Scientific). Chemical compounds used were as follows, at concentrations indicated in the figure legends: ER stress inducers, brefeldin A (BFA, 11861, Cayman Chemical), thapsigargin (TPG, 10522, Cayman Chemical) and tunicamycin (TM, 11445, Cayman Chemical); lysosomal inhibitors, bafilomycin A1 (Baf, 11038, Cayman Chemical, Ann Arbor, USA) and chloroquine (CHQ, C6628, Sigma-Aldrich); oxygen stress inducer, hydrogen peroxide (H_2_O_2_, H1009, Sigma-Aldrich); a chemical chaperone Tauroursodeoxycholate (TUDC, T0266, Sigma-Aldrich); and the PI3K inhibitor, wortmannin (Wort, 10010591, Cayman Chemical).

### Western blotting and ponceau S staining

Western blotting was performed as described previously^41^. In brief, treated cells, were washed with cold PBS for three times, harvested, and lysed in RIPA buffer (9806, Cell Signaling technology) containing protease inhibitor cocktail (P8340, Sigma-Aldrich) and 1% phosphatase inhibitor (P5726, Sigma-Aldrich). Protein concentrations were measured using the Bradford protein assay kit (T9310A, Takara, Shiga, Japan). Samples in a 2 × Laemmli sample buffer (161-0737, Bio-Rad, CA, USA) were heated at 95 °C for 5 min. Electrophoresis was performed in 5–20% SuperSep® Ace precast gels (Wako Pure Chemical Industries Ltd., Osaka, Japan). The transferred membranes were blocked with 5% skim milk (31149-75, Nacalai tesque, Kyoto, Japan) in PBS containing 0.1% tween 20 for 1 h at 20 °C, and then incubated overnight at 4 °C with the following Ab: anti-BECN1 (1:1000), anti-HSPA5 (1:3000), anti-LAMP1 (1:5000), anti-LAMP2 (1:4000), anti-MAP1LC3B (1:1000), anti-SQSTM1/p62 (1:2000), and anti-TUBA (1:5000). After treatment with secondary antibodies, membranes were visualized using an enhanced chemiluminescence detection system (32106, PIERCE, Rockford, IL, USA). Ponceau S staining (P7170, Sigma-Aldrich) of proteins transferred onto PVDF membranes was used for detecting protein levels in the cell culture media or in human serum samples.

### Isolation of primary human trophoblasts and cell culture

HchEpC1b cells, an EVT cell line, TCL1 cells, a third trimester trophoblast cell line, or BeWo cells, a choriocarcinoma cell line, were used in this study^42,43^. Cells were cultured in Roswell Park Memorial Institute (RPMI) 1640 medium (11875, GIBCO, MA, USA) for HchEpC1b cells or TCL1 cells, or Ham’s F12 medium (21127022, GIBCO) for BeWo cells, supplemented with 10% FBS, 100 U/ml of penicillin, and 100 µg/ml of streptomycin (15140, GIBCO) at 37 °C in a 5% CO_2_ atmosphere. Isolation and culture for primary human trophoblasts from term placentas was described previously^44^. The isolated cells were plated on multi-well plates at a density of 1.0 × 10^6^ cells/ml (100 μl/well for 96-well plates and 500 μl/well for 24-well plates), and cultured in Iscove’s modified Dulbecco’s medium (IMDM) supplemented with 10 ng/ml epidermal growth factor (GMP100-15, Pepro Tech, Rocky Hill, NJ, USA), 10 μM of Y27632 (a ROCK inhibitor, 08945, Nacalai tesque), 10% FBS, 100 U/ml of penicillin, and 100 µg/ml of streptomycin at 37 °C in a 5% CO_2_ atmosphere. The culture medium was changed at 12 h and 60 h after plating the cells, and then the cells were subjected to the experiments.

### Enzyme-linked immunosorbent assay (ELISA) measurements for PGF

After 96 h of culture, the culture media were harvested, and subjected to ELISA using the human GLB1/Beta-galactosidase ELISA kit (ELH-GLB1-1, RayBiotech, Peachtree corner, GA, USA). The range of the standard curve for PGF was 250–8,000 pg/ml. Generated signals were read at a wavelength of 450 nm with a microplate reader (1681135JA, iMark microplate reader, Bio-rad). All reactions were set in duplicates in the same plate, and at least three independent assays were set. Results were normalized to the cell numbers in each well.

### LAMP1/LAMP2 expression in culture media and cell viability assays

HchEpC1b cells were grown to sub-confluency in RPMI medium containing 2% FBS for 48 h. The culture media were harvested, centrifuged at 16,100 *g* for 10 min, and subjected to western blotting. Total protein levels in the media were determined by Ponceau S staining (P7170, Sigma-Aldrich). Cells harvested simultaneously, were stained with trypan blue (Sigma-Aldrich, T8154) and their viability was measured using Cellometer Auto T4 (Nexcelom Bioscience). There was no difference in cell viability upon DMSO (control) and TM treatment.

### Flowcytometry

Cells were harvested, washed twice with PBS containing 1% bovine serum albumin, and fixed in 4% paraformaldehyde for 15 min at room temperature. Cells were permeabilized and blocked in PBS containing 2% donkey serum and 0.1% Triton X-100, washed thrice with PBS, and incubated in dark at 4 °C for overnight with the following antibodies: LAMP1 (1:100 for cytoplasmic, 1:40 for surface staining), or LAMP2 (1:100 for cytoplasmic, 1:40 for surface staining). For LAMP1 or LAMP2 surface staining analysis, cells were incubated with the antibodies without permeabilization. Cells were washed, and subsequently stained with Alexa Fluor 488 donkey anti-mouse IgG (1:100; Molecular Probes) for 30 min at room temperature. A total of 10,000 events were recorded and analyzed using FACS Calibur flow cytometer (BD Biosciences) and WinMdi free software. Negative controls for each cell type were derived by incubating cells with purified mouse IgG1 immunoglobulin.

### Immunocytochemistry and lysotracker staining

Immunofluorescent staining was carried out as described previously^45^. Fixation and permeabilization were performed as described for flow cytometry^46^. Then, cells were labeled with the following primary Ab diluted with a Pierce immunostaining enhancer (ThermoFisher Scientific, USA): anti-LAMP1 (1:200), anti-SQSTM1 (1:200) or anti-MAP1LC3B (1:200). For negative controls, the primary Ab was replaced with normal rabbit IgG or normal mouse IgG. After washing out the primary Ab, cells were labeled with the secondary Ab: Alexa Fluor 488 donkey anti-mouse IgG (1:1000) or Alexa Fluor 555 donkey anti-rabbit IgG (1:1000); this was followed by nuclear staining with Hoechst33342 for 10 min. For lysosome detection, cells were treated with Lysotracker DND-99 (1 μM, Molecular Probes, L7528) for 40 min at 37 °C prior to fixation. Cells were observed using a confocal microscope (LSM700, Carl Zeiss, Oberkochen, Germany). Mean fluorescent intensity in each section was measured from at least 50 cells from ten randomly selected fields at 40X magnification using the ImageJ software (http://imagej.nih.gov/).

### Cell proliferation assay

Cell proliferation assay was performed with WST-1 (5015944, Roche, Basel, Switzerland). The absorbance of the medium, to which WST-1 was directly added, was measured after 2 h using a microplate reader (1681135JA, iMark microplate reader, Bio-rad) at a wavelength of 450 nm and at 650 nm as a reference. These experiments were performed in triplicates.

### Quantitative analysis of autophagosomes and autolysosomes

This assay was performed as previously reported^47,48^. For the quantitative analysis of autophagosomes and autolysosomes, the former was recognized by immunocytochemistry as MAP1LC3B puncta, and the latter as dots of MAP1LC3B that co-localized with or were surrounded by LAMP1 staining (a lysosomal marker). HchEpC1b cells, which were treated with BFA or TM, were treated with Baf at 10 nM for 2 h prior to fixation and visualization of the MAP1LC3B dots. In primary cells, Baf treatment was not performed for immunocytochemistry. The number of MAP1LC3B puncta or MAP1LC3B puncta juxtaposed with LAMP1 in a single cell was estimated by manual counting of thirty cells, using a confocal microscope. This experiment was performed at least three times.

### Statistical analysis

Results are presented as mean ± standard deviation. Kruskal-Wallis and Mann-Whitney (nonparametric) tests were used to compare the differences between groups. Values of *p* lower than 0.05 were considered significant.

## Supplementary information

Supplemental figures
