# Supplementary material for: Endoplasmic reticulum stress disrupts lysosomal homeostasis and induces blockade of autophagic flux in human trophoblasts
Source: Sci Rep. 2019 Aug 7;9:11466. doi: 10.1038/s41598-019-47607-5 (PMC6685987; doi:10.1038/s41598-019-47607-5)

Supplemental figures

Title: Endoplasmic reticulum stress disrupts lysosomal homeostasis and induces blockade of autophagic flux in human trophoblasts

Akitoshi Nakashima[1], Shi-Bin Cheng[2], Tae Kusabiraki[1], Kenichiro Motomura[3], Aiko Aoki[1], Akemi Ushijima[1], Yosuke Ono[1], Sayaka Tsuda[1], Tomoko Shima[1], Osamu Yoshino[1, 4], Haruhiko Sago[5], Kenji Matsumoto[3], Surendra Sharma[4], Shigeru Saito[1*]

Affiliations

[1] Department of Obstetrics and Gynecology, University of Toyama, 2630 Sugitani, Toyama, 930-0194, Japan.

[2] Departments of Pediatrics, Women and Infants Hospital of Rhode Island, Warren Alpert Medical School of Brown University, 101 Dudley street, Providence, RI, 02905, USA

[3] Department of Allergy and Clinical Immunology, National Research Institute for Child Health and Development, 2-10-1 Okura, Setagaya-ku, 157-8535, Tokyo, Japan

[4] Department of Obstetrics and Gynecology, Kitasato University School of Medicine, 1-15-1 Kitazato, Minami, Sagamihara, Kanagawa, 252-0374, Japan

[5] Center for Maternal-Fetal, Neonatal and Reproductive Medicine, National Center for Child Health and Development, 2-10-1 Okura, Setagaya-ku, 157-8535, Tokyo, Japan

*Corresponding author:
Prof. Shigeru Saito, 2630 Sugitani, Toyama, 930-0194, Japan,
Phone number: +81-76-434-7355 Fax number: +81-76-434-5036
Email address: s30saito@med.u-toyama.ac.jp

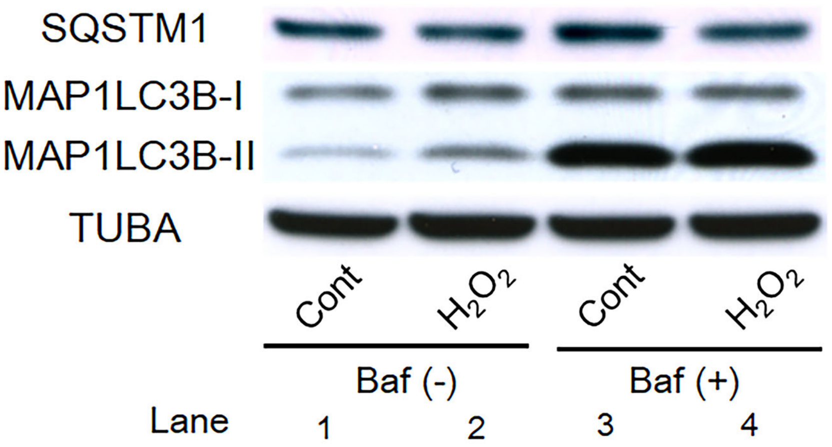

Supplemental Figure 1 Hydrogen peroxide, an oxidative stress inducer, did not affect autophagy in HchEpC1b cells.

Western blots of HchEpC1b cells cultured with 50 μM of hydrogen peroxide (H_2O_2) for 24 h with or without 10 nM of bafilomycin A1 (Baf) for 2 h at the end of culture showing: SQSTM1, MAP1LC3B (LC3) and TUBA.

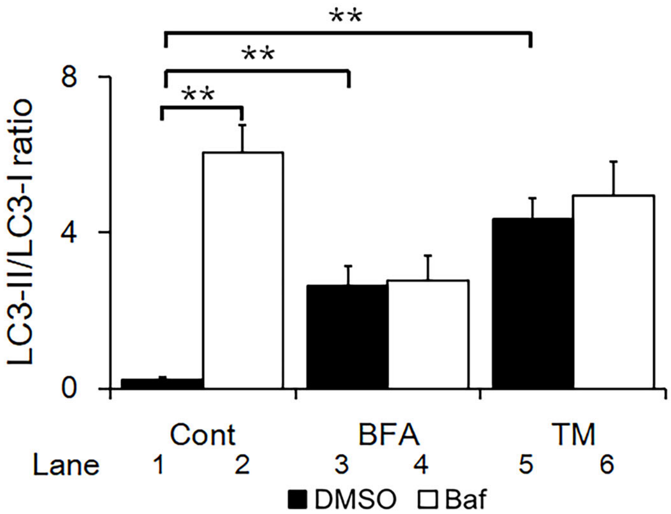

Supplemental Figure 2 The ratio of LC3-II/LC3-I in HchEpC1b cells with ER stress.
The graph shows the ratio of MAP1LC3B-II (LC3-II) to MAP1LC3B-I (LC3-I) in HchEpC1b cells, cultured with BFA or TM in the presence (white bars) or absence (black bars) of Baf. **, $p < 0.01$.

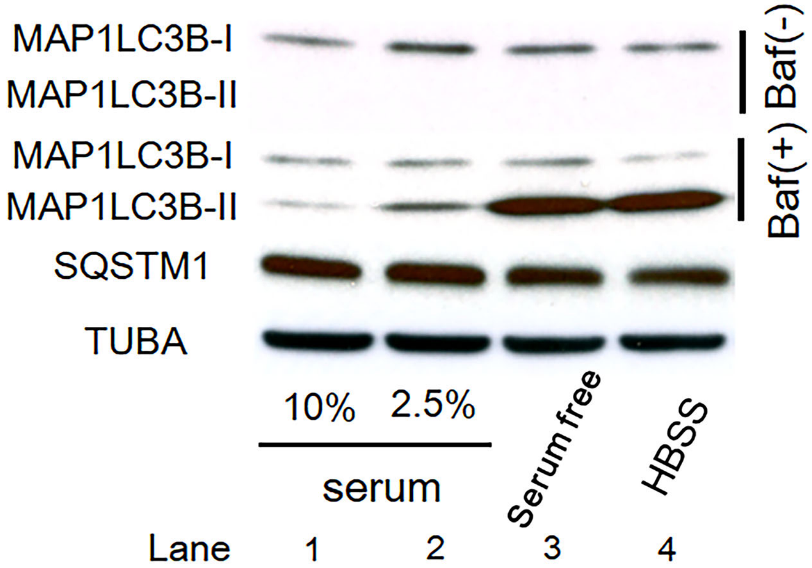

Supplemental Figure 3 Serum free media activated autophagy in HchEpC1b cells. Western blots of HchEpC1b cells cultured with 10% FBS, 2.5% FBS or serum free for 24 h, or HBSS for 2 h with or without 10 nM of bafilomycin A1 (Baf) for 2 h at the end of culture showing: SQSTM1, MAP1LC3B (LC3) and TUBA.

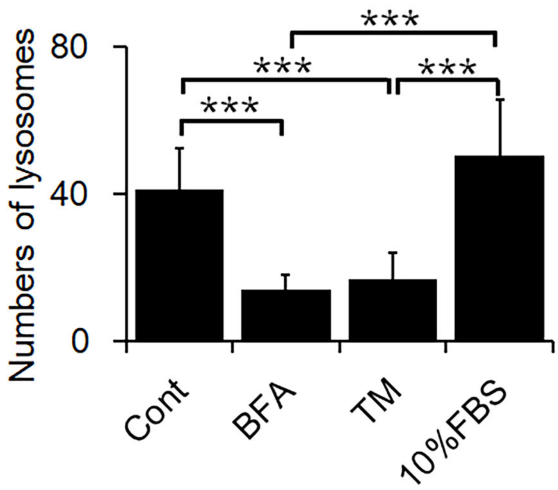

Supplemental Figure 4 The number of lysosomes was reduced by ER stress in HchEpC1b cells.

The graph shows the average numbers of lysosomes per cell (green dots in Fig. 1e) in HchEpC1b cells, which were cultured with DMSO (Cont) and serum free (SF), 500 ng/ml BFA and SF, 500 ng/ml TM and SF, or 10% FBS. Data is expressed as the mean ± S.D. ***, $p < 0.001$.

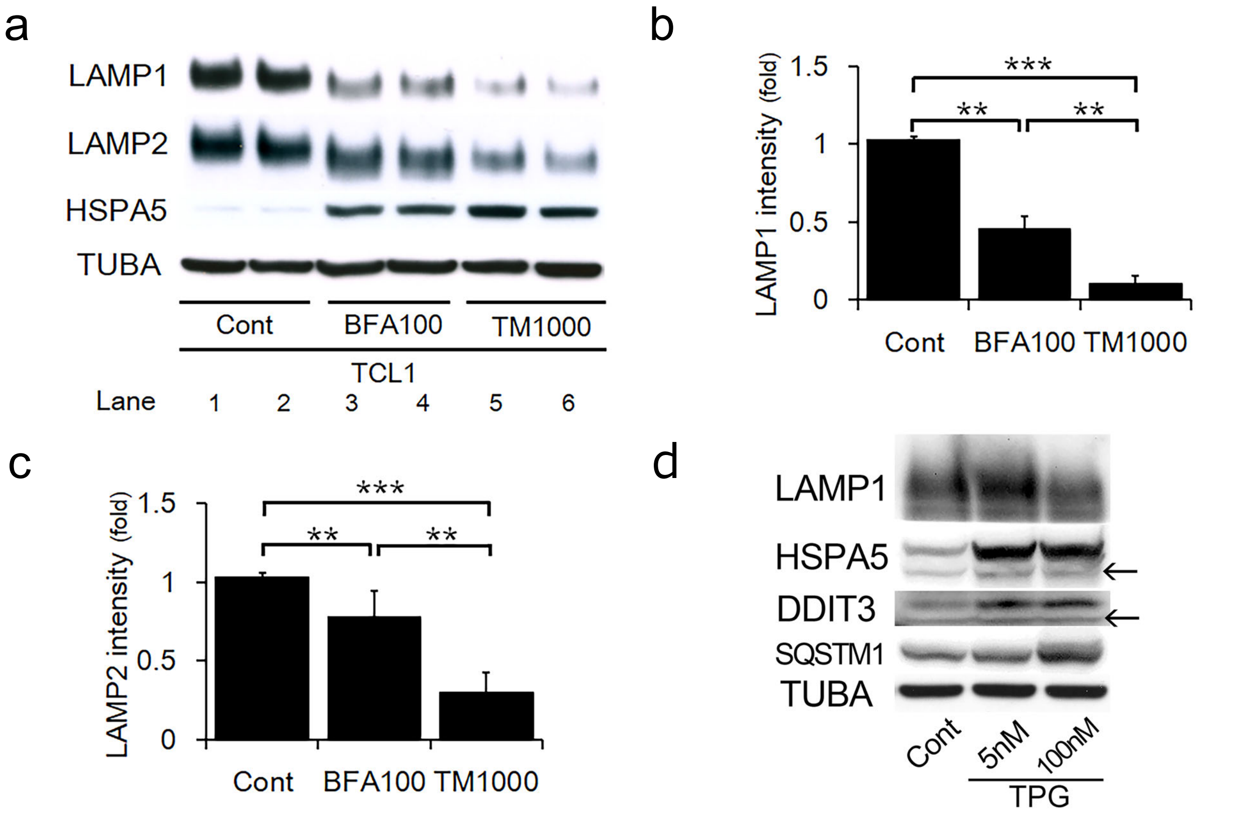

Supplemental Figure 5 ER stress suppressed the expression of LAMPs in TCL1 cells, a 3rd trimester cell line, or BeWo cells, a choriocarcinoma cell line.
(a) Western blots of TCL1 cells cultured with 100 ng/ml of brefeldin A (BFA) or 1,000 ng/ml of tunicamycin (TM) for 24 h showing: LAMP1, LAMP2, HSPA5 and TUBA. The graph shows the expression levels of LAMP1 (b) or LAMP2 (c) in TCL1 cells cultured with BFA or TM. Expression was normalized to TUBA levels. Data expressed as mean ± S.D.. (d) Western blots of BeWo cells cultured with 5 nM or 100 nM of thapsigargin (TPG) for 24 h showing: LAMP1, HSPA5, DDIT3, SQSTM1 and TUBA. Arrows indicated the non-specific bands. **, $p < 0.01$, ***, $p < 0.001$.

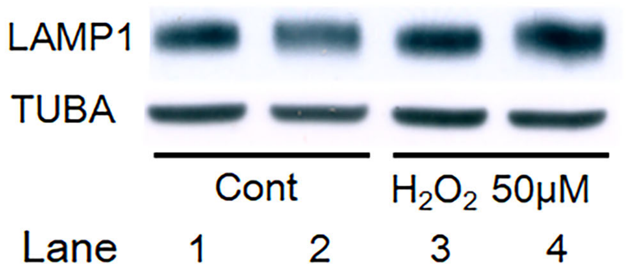

Supplemental Figure 6 Hydrogen peroxide did not affect LAMP1 expression in HchEpC1b cells.
Western blots of HchEpC1b cells cultured with 50 μM of hydrogen peroxide (H_2O_2) for 24 h showing: LAMP1 and TUBA.

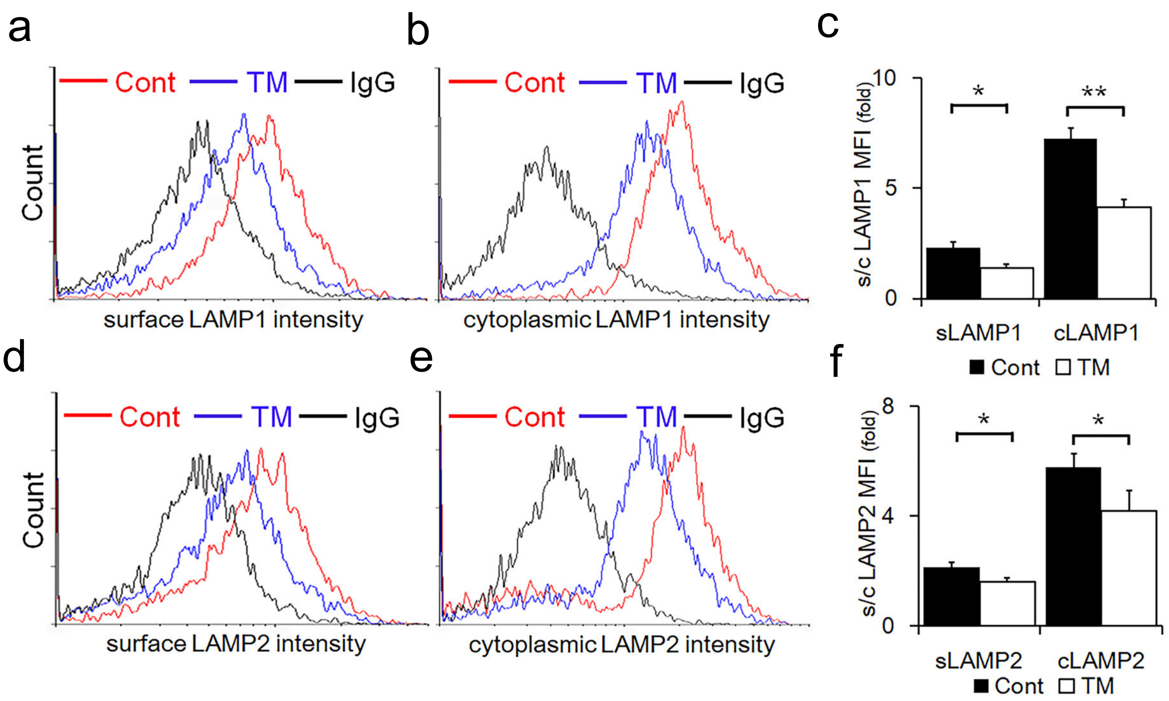

Supplemental Figure 7 ER stress downregulated cytoplasmic and surface LAMPs expression in HchEpC1b cells as assessed by flow cytometry.

Cytoplasmic and surface LAMPs expression in HchEpC1b cells treated with 500 ng/ml of TM, harvested with trypsin and analyzed by flow cytometry. Expression of surface LAMP1 (sLAMP1) is shown in (a) and cytoplasmic LAMP1 (cLAMP1) is shown in (b). (c) The graph shows the mean fluorescent intensity (MFI) of sLAMP1 or cLAMP1 in cells treated with DMSO (Cont, black bars) or TM (white bars) for 24 h. Expression of surface LAMP2 (sLAMP2) is shown in (d) and cytoplasmic LAMP2 (cLAMP2) is shown in (e). (f) The graph shows the MFI of sLAMP2 or cLAMP2 in the cells with DMSO (Cont, black bars) or TM (white bars) for 24 h. Red or blue lines correspond to the cells with DMSO (Cont) or TM. The black lines represent staining with appropriate control antibodies. Data are expressed as mean ± S.D.. *, $p < 0.05$, **, $p < 0.01$.

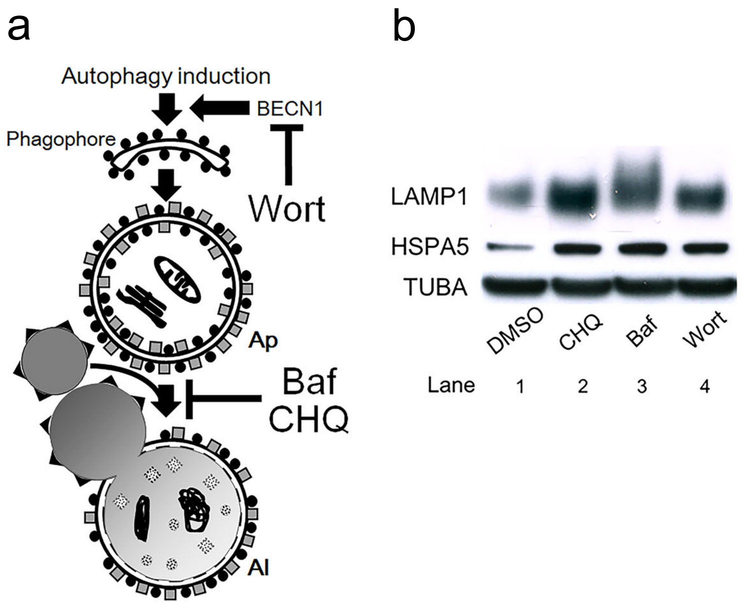

Supplemental Figure 8 Autophagy inhibitors induced ER stress in HchEpC1b cells.
(a) The schematic shows the effect of inhibitors on the autophagy machinery. Wortmannin (Wort) inhibits phagophore formation, while bafilomycin (Baf) or chloroquine (CHQ) treatment inhibits lysosomal functions. (b) Western blots of HchEpC1b cells cultured in 10 μM of CHQ, 20 nM of Baf, or 5 μM of Wort for 48 h showing: LAMP1, HSPA5 and TUBA.

Supplemental figure 9a Uncropped gel and blots for figure 1b, 2a, 2d, 5a, 5b, Supplemental Figure 1, 3, 5a, 5d, 6 and 8b.

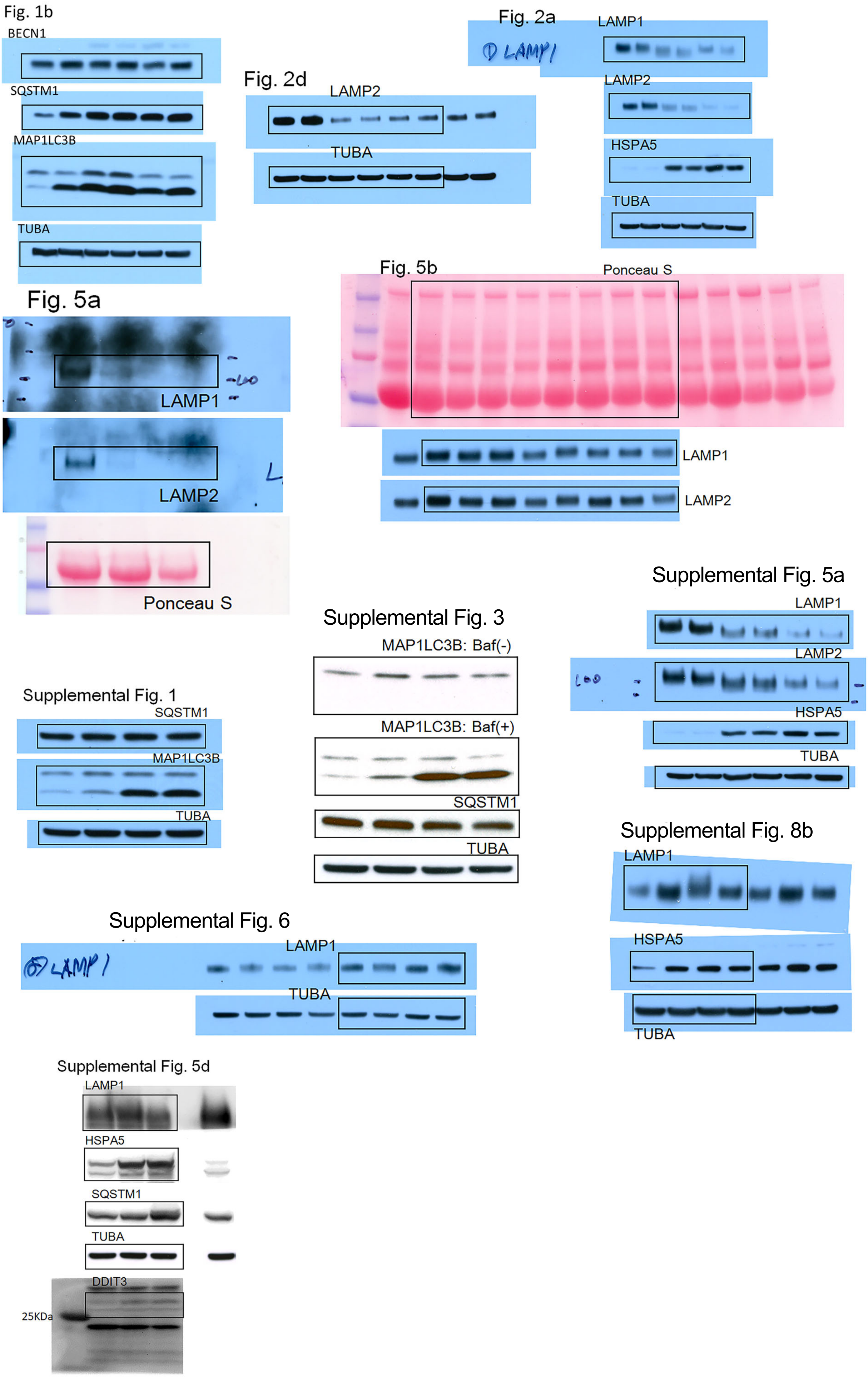

Supplement: Supplementary file 1 — Supplemental figures [file 41598_2019_47607_MOESM1_ESM.pdf]
